# Consumer Attitudes towards Local and Organic Food with Upcycled Ingredients: An Italian Case Study for Olive Leaves

**DOI:** 10.3390/foods9091325

**Published:** 2020-09-20

**Authors:** Maria Angela Perito, Silvia Coderoni, Carlo Russo

**Affiliations:** 1 Faculty of Bioscience and Technology for Food, Agriculture and Environment, University of Teramo, 64100 Teramo, Italy; 2 INRAE, ALISS, Université Paris-Saclay, 94205 Ivry-sur-Seine, France; 3 Department of Agricultural and Food Economics, Catholic University of the Sacred Heart, 29122 Piacenza, Italy; silvia.coderoni@unicatt.it; 4 Department of Economics and Law, University of Cassino and Southern Lazio, 03043 Cassino, Italy; carlo.russo@unicas.it

**Keywords:** olive leaves, organic, local, consumer attitude, up-cycled ingredients, by-products, generational differences

## Abstract

Food made with upcycled ingredients has received considerable attention in very recent years as a result of the need to both reduce waste and increase food nutritional properties. However, consumer acceptance of these novel foods is fundamental to their market uptake. This paper aims to assess the likelihood of the acceptance of food obtained from upcycled ingredients of olive oil productions and its association with some relevant recent consumption trends, such as organic food consumption and attention to food origin. In addition, particular attention is given to age group behaviors to appraise the differences between generations. Results suggest that, despite the negative influence of food technophobia, a core of sustainability-minded consumers seems to emerge that is interested in organic or local products, that could also favor the uptake of these novel food made with upcycled ingredients in the market. Results suggest that developing organic or “local” food products with upcycled ingredients can increase the probability of consumer acceptance.

## 1. Introduction

The olive tree is a central plant in the history of civilizations of the Mediterranean basin. It has historically been considered a sacred tree: in Genesis, the olive branch is a symbol of peace. Even in Greek Mythology, the olive branch was considered a symbol of peace and life. Ancient Romans rewarded valiant citizens with crowns of olive branches. In many cultures in the Mediterranean area, the olive branch is a symbol of justice. In addition, in the traditional culture of many Italian regions, olive leaves, as well as olive oil, have played the role of good luck against the evil eye [1]. Beyond the symbolic properties attributed to olive leaves, they have historically had a pharmacological role in Mediterranean countries and are widely used in traditional herbal medicine to prevent and treat various diseases [2,3].

This historical and anthropological excursus outlines the notion that olive leaves have almost never had a nutritional function in the tradition of Mediterranean areas, despite the importance of olive oil consumption [4,5]. The emergence of modern medicine and culture, however, has led to a loss of interest in olive leaves, and they have gradually become a processing waste for olive oil producers, with high disposal costs and no economic value.

Nevertheless, recent scientific literature has shown that olive leaves are rich in nutrients that could be used as by-products by the food industry to enrich food products with functional properties [6,7]. An essential point to understand the real market uptake of food enriched with food by-products that are not part of the traditional diet is to estimate consumer acceptance for such products, especially for food enriched with olive leaves.

Consumers consider food a complex good that contains both quality attributes as well as nutritional ones. Consumer acceptance is shaped by different factors, such as food habits and sociodemographic characteristics [8,9], food origin [5,10,11], the information that consumers access, and their trust in food production [12,13], the role of transparency [14], the range and prices of existing products [15,16,17,18,19], and the perception of health benefits and safety of food products [20,21].

In this context, the new objective of food production is no longer just food security but also the satisfaction of consumer needs and preferences with new products with both functional properties [22] and quality aspects (e.g., local, organic, etc.) [18,23]. In this context, the production of functional and nutritious food, obtained from upcycled ingredients, is a recent development [6,24,25,26,27].

Several studies referring to the use of unusual products in food production (i.e., insect products, cultured meat, etc.) report how new products can cause a strong food technophobia and neophobia in consumers [28,29,30,31,32]. Furthermore, studies on consumer acceptance of food derived from upcycled ingredients have found a negative impact of food technophobia and neophobia on the likelihood of acceptance [25,33]. However, interestingly, studies found that consumers may accord these products a premium status if promoted as a new food category akin to organic foods [34]. In fact, according to Bhatt et al. [34], consumers perceive “value-added surplus products” as having benefits for society and the individual.

The environmental sustainability of food production has become a matter of growing interest for consumers worldwide in recent years [35,36,37]. Often, environmental sustainability is understood by consumers as the preference to purchase organic food products, which are perceived as being healthier than conventional foods and better for the environment [18,38,39,40,41,42]. More recently, consumers have no longer been satisfied with the benefits offered by organic products only [43] but have been demanding domestic and local products as well [5,22,44,45,46]. In this respect, some studies suggest that consumers are interested in local food not only because they associate higher quality with these products but also for environmental friendliness, preference for their cultural roots [5,46], and support for the local economy [47] and local farmers [48].

Against this background, it seems to be of interest to analyze the possible impact of some frequent food consumption habits, such as buying organic products or giving importance to food origin in consumer acceptance of food derived from upcycled ingredients. In fact, foods obtained from upcycled ingredients of olive oil productions are new to the consumers, and their acceptance may be a problem despite their health or environmental benefits.

The main objective of the present study is to assess the association between the willingness to try food obtained from upcycled ingredients and consumer preferences for organic food and food origin. In particular, by estimating ordered probit models, we test if attributes, such as superior nutritional and/or environmental properties, of food with olive leaves are appealing to consumers. By doing so, we also investigate the possible market niches for these products.

In addition, to better understand consumer responses to food attributes, we run the analysis by age groups, as many studies have shown that elder and younger generations may actually differ in food preferences [49,50].

## 2. Materials and Methods

To investigate the association between the willingness to try food obtained from upcycled ingredients and the preference for organic and food origin, we surveyed a sample of 852 Italian consumers. The core of our analysis was an ordered probit regression [51] of a discrete variable measuring the willingness to try food with upcycled ingredients on a set of regressors, including demographic variables, measures of consumer environmental responsibility, technophobia, and concerns for product origin.

Data were collected through a web-based survey administered in Italy between April 2018 and April 2019 with a convenience approach. Participants were reached via different social media networks, which is becoming a more popular means of reaching participants in social sciences research for both convenience and inclusion reason [52]. In fact, the use of the Internet makes it as convenient as possible for participants to take part in the survey and allows reaching a high number of participants from all Italian regions. In particular, the information was then posted on Twitter, LinkedIn, and Facebook pages. As sampling in Internet research studies is not random and could generate selection bias, to minimize this possible problem, we posted the questionnaire on pages and online groups with a general target audience (e.g., web pages of Italian radio programs).

Before answering the questions, participants were briefly informed about the research project that motivated the survey.

Respondents were given a short, four-section questionnaire. Section 1 collected the demographic information, Section 2 assessed the respondent’s attitudes toward the covariates of interest (organic food and food origin), Section 3 investigated the respondent’s technophobia [29,30], and Section 4 asked the respondent’s willingness to try food obtained from by-products [33]. Descriptive statistics of the sample and the questionnaire are presented in Table 1.

To lessen collection cost and maximize the response rate, we minimized the number of items in the questionnaires and drafted questions as five-point Likert scales. The final design was extremely parsimonious and included 11 questions (see Table 1).

We measured the attitude toward organic food, asking respondents to state the frequency of their purchase of organic food on a 5-point scale (from never to always). To limit the possible self-representation bias, we referred to a specific action (buying organic food) instead of asking to report the attitude directly. This approach was possible because Italian consumers, on average, are familiar with organic products, given the sharp increase in organic consumption in the last decade (in 2017, 78% of Italian family had bought organic food at least once, and 48% had bought them at least once a week [53]).

Consumer perception of food origin was blurred mostly because of the overlapping of several different concepts, such as local food, typical food, or food safety. As a consequence, the questionnaire asked to report the importance of product origin in the food purchase decision.

Food technophobia (or food technology neophobia) is defined as consumers’ fear, dislike, or avoidance of novel food technology [29]. Perito et al. [33] found that it is a key driver limiting consumer acceptance of food with olive by-products. As a consequence, we included it as a control variable in our empirical investigation.

Technophobia is a complex attitude to measure. Several contributions in the literature proposed scale measures [29,54,55]. In this paper, we adopt the approach proposed by Perito et al. [33]. The measure is based on three statements:

NNNT: There is no need for new food technologies because there are so many types of foods;
NTOR: The benefits associated with innovative food technologies are often overestimated;
NTLQ: New food technologies reduce the natural quality of foods.

Respondents were asked to agree or disagree with the statements on a 5-point Likert scale from 1 (strongly disagree) to 5 (strongly agree). Cronbach α of 0.78 confirmed that the construct items were consistent. We defined the technophobia index (TFI) as the average of the scores of the three variables.

Finally, the questionnaire asked respondents to report their willingness to try food with olive leaves as upcycled ingredients on a 5-point scale from 1 (No, Absolutely) to 5 (Yes, Absolutely). Because Perito et al. [33] identified environmental and nutritional concerns as key drivers of consumer acceptance, we conditioned the answer to two situations: A) the upcycled ingredients of olive leaves has superior nutritional properties (TNUT), and B) the upcycled ingredients reduces the environmental impact of food production and consumption (TENV). In this way, we provide useful insights to novel food researchers willing to market food with upcycled ingredients and different characteristics. Our hypothesis is that products with different attributes (nutritional or environmental) may be appealing to different consumers.

To investigate the association between consumer willingness to try food enriched with upcycled ingredients, organic food, and food origin, we ran a regression of the dependent variables TNUT and TENV on a vector of demographic variables (gender, age, education, and employment status), the technophobia index (TFI), and the attitudes regarding organic food (ORGANIC) and product origin (ORIGIN). Given the discrete nature of the dependent variables, we used an ordered probit model. In fact, in our case, the dependent variables were ordinal, but not continuous in the sense that the metric used to code the variables was substantively meaningful. For instance, the 5-point scale adopted to measure the dependent variable assigned the numerals to the categories but the metric underlying response identification was not necessarily the same as the linear metric relating the numerals. In other terms, the difference between 0 and 2 on the coded responses may be quite different from the difference between 2 and 4. A widely used approach to estimating models of this type is an ordered response model. The basic assumption of such models is that there is a latent continuous metric underlying the ordinal responses observed by the analyst.

The model estimated, assuming that the values of TNUT and TENV were the observable outcome of latent variables, is the following:

(1) $${\hat{Y}}_{h}=f\left(GENB,\;AGE,\;EDUCATION,\;EMPLOYMENT,\;TFI,\;ORGANIC,\;ORIGIN\right)$$

where h = N, E; GENB is a binary variable that is equal to 1 if the respondent is female; AGE is the respondent’s age; EDUCATION is a categorical variable with four entries: elementary school, middle school, high school, and college, depending on the respondent’s degree; EMPLOYMENT is a five-entry categorical variable equal to the worker (if the respondent is employed or self-employed), unemployed, student, homemaker or retired, depending on the respondent’s status.

Regarding the variable AGE, a further refinement is then introduced as a separate regression for each age group has been conducted to exploit the large sample size of our survey.

## 3. Results

### 3.1. Sample Description

The sample was composed of 852 respondents aged between 18 and 90 years old, with an average of 37 years and six months. Sixty-five percent of respondents were female. Table 2 reports the social and demographic characteristics of the sample.

Table 3 illustrates the distribution of the variables. The majority of respondents stated that they buy organic food at least “seldom”. Eighty-seven percent of respondents stated that product origin was an important or very important driver of food purchase decisions (Table 3). Such a high figure is explained by the broad meaning of the term origin that in the consumers’ mind, is associated with local food, Italian food, typical products, and, in a broader sense, even to food safety. The value origin and the specific reputation for very local products are extensively documented in the literature [11]. Region of origin evokes tradition, habits, culture, and so on, and these aspects directly influence preference for a regional product [56,57,58].

**Figure 1:**
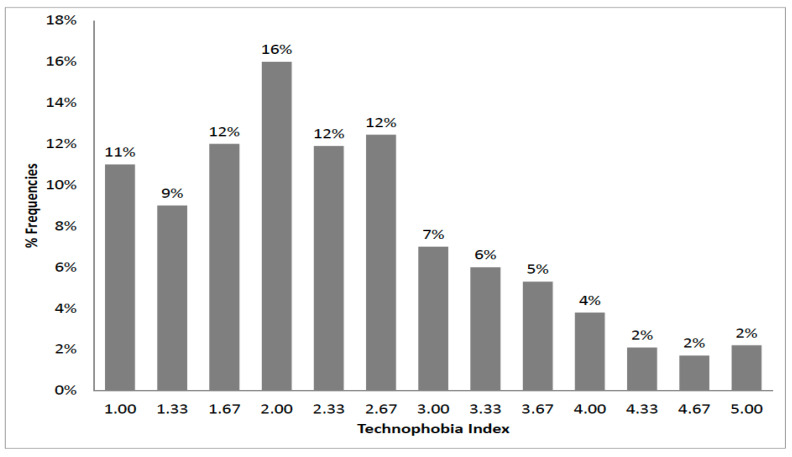
Distribution of the Technophobia Index (TFI).

Table 3 reports the distribution of the three original variables (NNNT, NTOR, and NTLQ), and Figure 1 illustrates the distribution of TFI.

**Figure 2:**
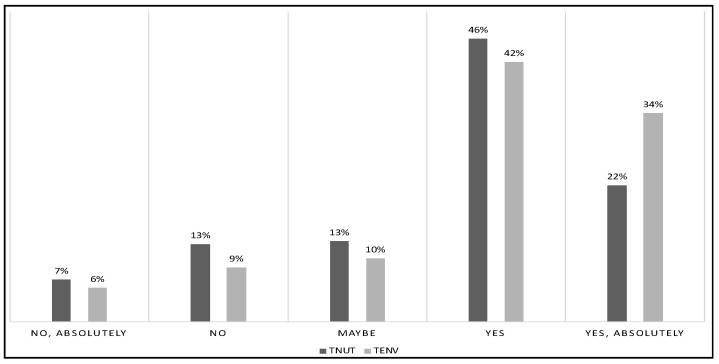
Willingness to try food containing upcycled ingredients with superior nutrition properties (TNUT) or lower environmental impact (TENV).

Our respondents reported different willingness to try food with upcycled ingredients of olive leaves based on environmental and nutritional concerns. Our finding is that products with different attributes (nutritional or environmental) can be appealing to different consumers. Figure 2 illustrates the distribution of the two variables.

Variables TNUT and TENT are not independent. A Fisher’s exact test rejected the null hypothesis of independence at a 99% confidence level. Table 4 reports Pearson’s standardized residuals from the contingency table, showing that the diagonal elements were positive, while the off-diagonal ones were mostly negative. (Pearson’s standardized residuals are computed by subtracting the expected frequency in a given cell under the null hypothesis of independence from the actual observed frequency and then dividing by the square root of the expected frequency.) This result suggests a positive association and that the two drivers of consumer acceptance do not offset each other on average. The result is of particular importance because it shows that the two drivers may be pursued at the same time.

### 3.2. Model Results

Table 5 reports the results of the estimation of the model in Equation (1). An χ_2_ test on the joint significance of the coefficients of demography, education, and working status variables failed to reject the null hypothesis that all coefficients were jointly equal to zero (*p*-values 0.290 and 0.597 in the TNUT and TENV regressions, respectively) (The *p*-values of the χ^2^ on the joint significance of specific groups of variables (demography, education, employment status) are reported in Table 3.)

Technophobia’s coefficient was negative and statistically different from zero at a 95% confidence level in both regressions. High values of TFI were associated with a higher probability of being absolutely unwilling to try olive by-product food. Table 6 reports the marginal probabilities.

An χ_2_ test failed to reject the hypothesis that ORGANIC did not affect TNUT. Instead, we detected a statistically significant association with TENV (95% confidence level). Holding all other variables constant, buyers of organic food are expected to be more willing to try food with olive by-products if the consumption is beneficial for the environment.

In addition, respondents considering product origin an important or very important issue in food choice are more likely to be very willing to try food with olive by-products. The results hold in both regressions.

### 3.3. Differences across Generations

The regressions in Table 3 failed to reject the null hypothesis that the conditional expectations of TENV and TNUT were unaffected by the respondent’s age. However, several studies have shown that older generations show different food preferences to younger generations [49,50,59]. To investigate this point further, we exploited the large sample size of our survey to run separate regression for age groups.

**Figure 3:**
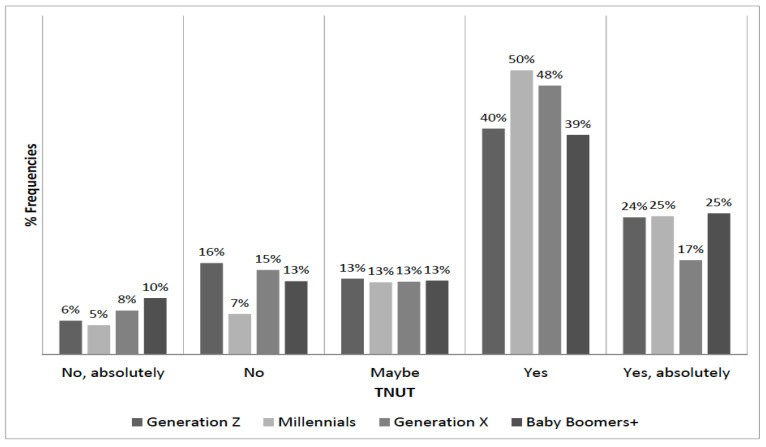
Distribution of TNUT by generations.

**Figure 4:**
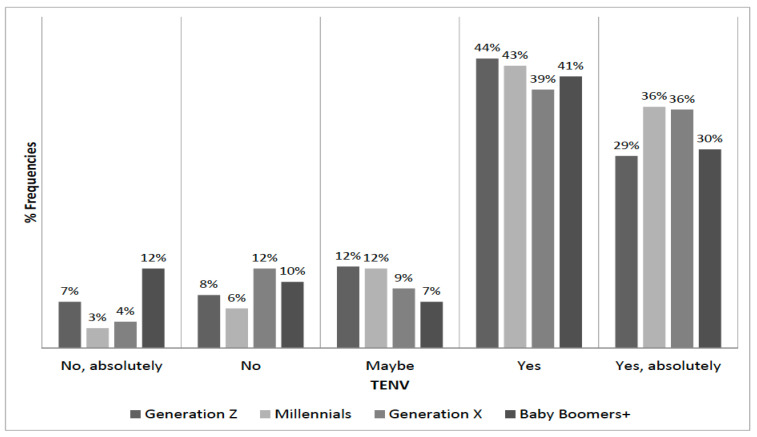
Distribution of TENV by generations.

We split the sample into four age groups: Generation Z (age between 18 and 24), Millennials (or Generation Y, age between 25 and 39), Generation X (between 40 and 54), and Baby Boomers+ (age 55 or above). Figure 3 and Figure 4 illustrate the distribution of TNUT and TENV by age group, respectively. An χ2 test failed to reject the null hypothesis of independence between TNUT and age groups at a 95% confidence level (*p*-value 0.057). Instead, the independence of TENV and age group was rejected (*p*-value 0.01).

Table 7 and Table 8 report the outcome of the regressions of TNUT and TENV by generation groups, respectively. For the reader’s convenience, the results of the χ_2_ test on the joint significance of coefficients of groups of variables (Demography, Education, Employment status, Organic, and Origin) are summarized in Table 9.

The analysis by age group showed that the drivers of willingness to accept food with upcycled ingredients were not monotonic with respect to the respondent’s age. Each generation had distinctive characteristics that were not necessarily similar to the next age group. This result explains why the coefficients of the AGE and AGE2 variables were not statistically different from zero in the full sample regression and suggests that the ensemble of beliefs driving the behavior of a generation may be defined in contrast to the previous generation.

Generation Z’s (age 18–24) acceptance of food with olive by-products and improved nutrition characteristics was driven mainly by technophobia. Working status affects decisions, with students showing a higher willingness to try than unemployed respondents (An χ^2^ test rejected the null hypothesis that the coefficients w2 and w3 were equal with *p*-value 0.006.). The coefficient of the quadratic form of the variable AGE on TNUT was statistically different from zero, suggesting that preferences within Generation Z were not homogeneous. Generation Z’s decisions to try food with upcycled ingredients that reduce environmental impact were affected by technophobia and working status. A positive association with organic purchase was found as well.

The willingness of Millennials (age 25–39) to try upcycled ingredients with improved nutrition attributes was driven by technophobia alone. Decisions regarding environmentally friendly by-products were driven by technophobia, education, and organic purchase. The importance of product origin was not associated with the dependent variables.

The drivers of Generation X’s (40–54) decisions regarding TENV and TNUT were similar. The coefficients of Employment status variables, TFI, and ORIGIN were statistically different from zero in both regressions at a 95% confidence level. ORGANIC was statistically significant at a 95% confidence level in the TENV regression and at a 90% confidence level in the TNUT regression.

Finally, the age group Baby Boomers+ (55+) exhibited different behaviors depending on whether the upcycled ingredients were associated with improved nutrition or environmental responsibility. In the former case, technophobia was the only driver. In the latter case, ORGANIC and ORIGIN variables were associated with higher values of willingness to try.

## 4. Discussion

Among the greatest challenges the world faces today are how to ensure that a growing global population has access to enough healthy food and how to reduce food loss and waste. Rethinking the food system and implementing circular resource management systems will help mitigate the effects of food production on the environment and limited availability of resources [60].

Waste valorization has been defined by Arancon et al. [61] as the process of converting waste into more useful products. For example, the olive tree pruning produces 25 Kg of waste biomass for each tree annually, and approximately 25% is leaves [62]. Olive leaves are rich in phenolic compounds [6], and the food sector should use them to produce value-added products [25]. However, consumer acceptance of these products is fundamental to their market uptake.

This study aimed at answering two main research questions: What interest do consumers have in food products enriched with waste-to-value food? Which variables are important predictors of consumer willingness to buy food products enriched with waste-to-value food? Previous results suggest that, although the production of foods with up-cycled ingredients is technically feasible [6,7], carefully-designed marketing campaigns are necessary to ensure consumer acceptance and, ultimately, economic success [38,45]. Aschemann-Witzel and Peschel [27], analyzing how Danish consumers react to the use of by-products in some food products, indicated that specific brand, design, and specific quality information on these new ingredients could improve consumer attitudes towards the “waste-to-value” products.

Our study is useful to highlight what specific consumer profiles may be targeted for marketing campaigns. Environmentally responsible organic consumers, in fact, are likely to be an important niche for food with olive by-products. In the sample analyzed, buyers of organic food were expected to be more willing to try this novel food if it was more beneficial for the environment. This result is quite well known in the literature and might be explained with the special concern for the environmental aspects that organic buyers show [25,63,64,65].

The preference of organic consumers for such novel environmentally sustainable products is very important, considering the current market trends. In fact, nowadays, organic food is not a niche market anymore, accounting for approximately 3% of the total value of the agri-food sector [66,67]. In particular, Italy is ranked sixth in the world among the countries with the largest area cultivated with organic farming methods [68,69]. Furthermore, environmentally sustainable consumption is gaining importance in the market, with consumers showing higher interest in the impacts on natural resources of their food purchases [23].

On the contrary, nutritional attributes were not appealing for environmentalist consumers, as the lack of association between organic purchase and acceptance of upcycled food with superior nutritional properties showed. This finding seems in line with Grasso and Assioli [26], who analyzed three different groups of consumers and the group called “environmentalist”, more interested in the environment, had the lowest rejection towards upcycled sun-flower flour in biscuits.

Another result deserving particular attention is one of product origin. In fact, respondents considering product origin an important or very important issue in food choice were more likely to be willing to try food with olive by-products. As product origin is a very important driver of consumer choice in the Italian market [5], this result is of interest because it suggests that there could be a marketing potential for local food made with upcycled ingredients. In fact, our results confirm that the origin of the by-product may mitigate the food technophobia, and origin information on the olive by-products can increase consumer acceptance and preference for food with upcycled ingredients.

Interesting insights on the consumer characteristics can be derived from the differences across the generations analyzed. In fact, the drivers of willingness to accept food with upcycled ingredients were not the same with respect to the respondent’s age. Each generation had distinctive characteristics that were not necessarily similar to the next age group. This result explains why the coefficients of the AGE and AGE2 variables were not statistically different from zero in the full sample regression and suggests that the ensemble of beliefs driving the behavior of a generation may be defined in contrast to the previous generation.

For the youngest Z Generation, technophobia and working status were the relevant drivers in determining acceptance of food with by-products with improved nutrition characteristics or with reduced environmental impact. In this latter case, a positive association with organic purchase and a slight importance of a product’s origin was found as well, confirming the general result regarding organic consumers and product origin discussed above.

For Millennials, technophobia alone seemed to drive the acceptance of food with upcycled ingredients with improved nutrition attributes, while decisions regarding environmentally friendly by-products were driven by technophobia, education, and organic purchase. Interestingly, a distinctive characteristic of Millennials, compared to other age groups, was that the importance of product origin was not associated with their willingness to accept, thus highlighting a different behavior of that generation regarding this product’s feature.

Generation X seemed, instead, to show more similar and coherent preferences regarding both food with upcycled ingredients with improved nutrition attributes and lower environmental impact. For this group of consumers, technophobia, product’s origin, and organic consumption were all relevant in affecting their purchase intentions.

Baby Boomers+, instead, showed different preference structures. For food with upcycled ingredients with improved nutritional properties, technophobia seemed to be the only driver. For environmentally sustainable food with up-cycled ingredients, the willingness to accept was mainly driven by product origin and organic preferences.

Differences across generations allow even better targeting of market delivery of the product, focusing the attention on the specific driver of each age group segment. In fact, results, if confirmed by further surveys in other countries and with larger and more representative samples, suggest that different age groups respond differently to product characteristics and they could be better targeted with more specific and ad-hoc campaigns. For example, the aspect of a product’s origin seemed not to be relevant in determining the Millennials acceptance for food with up-cycled ingredients, and thus it could be argued that to target such consumers, the attention should be paid to better presenting the “low technological component” in the production process, rather than the local origin of the product to the consumer.

A final consideration should be given to the fact that, at the time of the survey, the proposed novel product was not yet available in the market. This aspect might represent a weakness in the proposed analysis. The fact that consumers were not able to test or see the product could have influenced their replies. However, the results presented here could be useful in the market launch of these products as they target consumers who have shown willingness to purchase such products (e.g., consumers of organic and local products) according to their specific age group.

## 5. Conclusions

Consumer’s general perception of the use of upcycled ingredients for food production is that the food industry tries to save money with inputs obtained at a lower cost [70]. Consumers accept many by-products for pharmaceutical use because they are rich in healthy components. The acceptance of by-products used for food production is a more complex matter because of the influence of certain levels of food neophobia or technophobia that might hamper the uptake of such products.

The present study confirmed this general influence as both the complete sample and all age groups of consumers demonstrated that technophobia was negatively influenced by the probability of accepting food enriched with olive oil by-products [33]. However, two major determinants of consumption of food made with upcycled ingredients emerged: organic consumers are more likely to accept this novel food and, also, consumers who consider product origin an important or very important issue in food choice are more likely to be willing to try food with olive by-products. The impact of such aspects slightly differs when referred to different product’s characteristics. When we consider products with superior nutrition properties, the association with origin attributes was stronger, while when looking at food with lower environmental impact, the consumption of organic food seemed to be highly associated with the acceptance of the novel food.

This latter aspect also emerged if we look at the results for the generation groups. In all the age groups, the consumption of organic products was positively associated with a likely acceptance of food made with upcycled ingredients, which show a lower environmental impact.

Results here presented would thus suggest that there could be a core of consumers interested in organic or local products, that could also favor the uptake of these novel foods made with upcycled ingredients in the market. Marketing policies are of great importance in that sense because indicating the benefits these foods could bring to health and the environment clearly in the label should help to deliver novel food to the greater public. According to the results of this study, developing organic or “local” varieties of food with upcycled ingredients might increase the probability of consumer acceptance.

Our study adds another piece to the puzzle of the research into upcycling or waste-to-value products in the area of food where studies are yet scarce [25,26,27,33].

However, this manuscript has two main limitations. First, the sample analyzed in this study is not representative of the whole Italian population. However, given the size of the sample, the relationships between the variables analyzed and the positive purchase intention eventually expressed remain valid and allow us to obtain interesting results. Second, as also reported by Grasso and Assioli [26], because upcycled ingredients for food products are not on the market yet and there is not an appropriate definition of these products, our study might suffer from hypothetical bias, which could have affected the estimation of consumer acceptance.

Future research is needed to confirm our results in other countries and using different products and/or upcycled ingredients. In particular, an experimental approach can be used to overcome the hypothetical bias.

## Figures and Tables

**Table 1:** Questionnaire and descriptive statistics of variables (*n* = 852).

Questionnaire	Label Variable	Scale	Mean				
Gender	GEN		Male			Female	
			35.33			64.67	
Age	AGE		37.56				
Education	EDU		Elemen.	Middle		High S.	College
			0.35	2.35		37.09	60.21
Employment	EMPL		Worker	Unempl.	Student	Homem.	Retired
			59.04	5.87	28.29	2.70	4.11
Frequency of the purchase of organic food	ORGANIC	5-point scale	Never	Seldom	Somet.	Often	Always
			46.48	23.59	5.99	14.44	9.51
Is food Origin (e.g., local/typical product) important when shopping for food?	ORIGIN	5-point scale	Abs. Not	Not	Indiff.	Yes	Abs. Yes
			2.46	3.76	7.28	38.97	47.53
There is no need for new food technologies because there are so many types of foods.	NNNT	5-point scale	Abs. Disag.	Disagr.	Indiff.	Agree	Abs. Agree
			39.20	35.68	10.09	10.68	4.34
The benefits associated with innovative food technologies are often overestimated	NTOR	5-point scale	Abs. Disag.	Disagr.	Indiff.	Agree	Abs. Agree
			18.31	35.56	18.19	20.07	7.86
New food technologies reduce the natural quality of foods	NTLQ	5-point scale	Abs. Disag.	Disagr.	Indiff.	Agree	Abs. Agree
			23.83	36.97	15.96	15.61	7.63
*I am willing to try food with upcycled ingredients of olive leaves if:*							
it has superior nutritional properties	TNUT	5-point scale	Abs. Not	Not	Indiff.	Yes	Abs. Yes
			6.81	12.56	13.03	45.66	21.95
it reduces the environmental impact of food production and consumption	TENV	5-point scale	Abs. Not	Not	Indiff.	Yes	Abs. Yes
			5.52	8.80	10.21	33.57	41.90

**Table 2:** Sample demographics characteristics.

Education	% Freq.	Employment Status	% Freq.	Gender	% Freq.	Age	% Freq.
Elementary school	0.35	Worker	59.04	Male	35.33	Generat. Z (18–24)	25.23
Middle School	2.35	Unemployed	5.87	Female	64.67	Millennials (25–39)	31.34
High School	37.09	Student	28.29			Generat. X (40–54)	30.99
College	60.21	Homemaker	2.70			Elder Gen. (55+)	12.44
		Retired	4.11				
Total	100.00	Total	100.00	Total	100.00	Total	100.00

**Table 3:** Distribution of selected variables.

BUYBIO			ORIGIN				NNNT		NTOR		NTQL	
Scale	N	%	Scale	N	%	Scale	N	%	N	%	N	%
Never	396	46.5	Absol. not important	21	2.5	Strongly disagree	334	39.2	156	18.3	203	23.8
Seldom	201	23.6	Not important	32	3.8	Disagree	304	35.7	303	35.6	315	37.0
Sometimes	51	6.0	Indifferent	62	7.3	Not agree nor disagree	86	10.1	155	18.2	136	16.0
Often	123	14.4	Important	332	39.0	Agree	91	10.7	171	20.1	133	15.6
Always	81	9.5	Very important	405	47.5	Strongly agree	37	4.3	67	7.9	65	7.6
Total	852	100.0	Total	852	100.0	Total	852	100.0	852	100.0	852	100.0

Note: BUYBIO: Do you buy organic food? ORIGIN: How important is product origin when deciding your food purchase? NNT: There is no need for new food technologies because there are so many types of foods; NTQR: The benefits associated with innovative food technologies are often overestimated; NTQL: New food technologies reduce the natural quality of foods.

**Table 4:** Pearson’s standardized residuals from a two-way table of superior nutrition properties (TNUT) and lower environmental impact (TENV).

	TENV				
TNUT	No, absol.	No	Maybe	Yes	Yes, absol.
**No, absolutely**	17.8	0.8	−2.0	−3.1	−3.1
**No**	0.5	12.9	−0.3	−3.0	−3.3
**Maybe**	−1.3	−0.6	6.1	−0.5	−2.0
**Yes**	−4.4	−4.1	−0.7	6.1	−2.5
**Yes, absolutely**	−2.9	−3.8	−2.3	−4.4	9.4

**Table 5:** Regression results.

		TNUT			TENV		
		*Coeff*	*Std. Error*	*p-Value*	*Coeff*	*Std. Error*	*p-Value*
***Demography***				*0.971*			*0.437*
Female respondent	Genb	−0.012	0.076	0.872	−0.035	0.080	0.658
Age of respondent	Age	0.009	0.029	0.750	0.041	0.027	0.130
Age squared	age2	0.000	0.000	0.796	0.000	0.000	0.164
***Education***				*0.098*			*0.857*
Elementary school							
Middle school	ed2	−0.816	0.612	0.183	0.106	0.396	0.789
High school	ed3	−1.081	0.590	0.067	0.188	0.370	0.611
College	ed4	−0.915	0.588	0.120	0.231	0.365	0.526
***Working status***				*0.167*			*0.358*
Worker							
Unemployed	w2	0.135	0.174	0.439	0.031	0.182	0.867
Student	w3	0.224	0.167	0.180	0.216	0.177	0.221
Homemaker	w4	0.582	0.275	0.034	0.436	0.262	0.096
Retired	w5	−0.006	0.298	0.983	−0.031	0.264	0.906
***Organic***				*0.145*			*0.000*
Never							
Seldom	bb2	−0.209	0.098	0.033	−0.218	0.099	0.028
Sometimes	bb3	0.132	0.169	0.436	0.583	0.147	0.000
Often	bb4	−0.105	0.104	0.314	0.504	0.120	0.000
Always	bb5	0.024	0.140	0.866	0.817	0.160	0.000
***Origin***				*0.006*			*0.000*
Absol. not important							
Not important	bo2	0.177	0.368	0.631	0.116	0.365	0.751
Indifferent	bo3	0.559	0.323	0.084	0.460	0.328	0.161
Important	bo4	0.725	0.310	0.019	0.812	0.315	0.010
Very important	bo5	0.769	0.310	0.013	0.897	0.317	0.005
***Technophobia***	TFI	−0.368	0.046	0.000	−0.301	0.045	0.000
Observations		852			852		
Pseudo R^2^		0.052			0.076		
Wald chi^2^		115.38		0.000	152.01		0.000

Note: Figures in Italic font report the *p*-values of a test on the joint significance of the regression coefficient of the corresponding group of variables (demography, education, working status, organic, origin).

**Table 6:** Marginal probabilities.

				TNUT					TENV		
	Variables	TNUT = 1	TNUT = 2	TNUT = 3	TNUT = 4	TNUT = 5	TENV = 1	TENV = 2	TENV = 3	TENV = 4	TENV = 5
	Female respondent	0.001	0.002	0.001	−0.001	−0.003	0.003	0.004	0.004	0.002	−0.013
**Demography**	Age of respondent	−0.001	−0.001	−0.001	0.001	0.003	−0.003	−0.005	−0.004	−0.002	0.015
	Age squared	0.000	0.000	0.000	0.000	0.000	0.000	0.000	0.000	0.000	0.000
	Elementary school										
	Middle school *	0.153	0.122	0.041	−0.160	−0.155	−0.007	−0.012	−0.011	−0.008	0.039
**Educational**	High school *	0.150	0.158	0.082	−0.123	−0.266	−0.014	−0.022	−0.020	−0.013	0.067
	College *	0.088	0.126	0.087	−0.029	−0.272	−0.018	−0.027	−0.024	−0.011	0.081
	Worker										
	Unemployed *	−0.013	−0.020	−0.014	0.007	0.040	−0.002	−0.004	−0.003	−0.002	0.011
**Employment**	Student *	−0.022	−0.033	−0.022	0.012	0.065	−0.015	−0.024	−0.023	−0.016	0.078
	Homemaker *	−0.039	−0.072	−0.061	−0.024	0.196	−0.023	−0.042	−0.044	−0.058	0.166
	Retired *	0.001	0.001	0.001	0.000	−0.002	0.002	0.004	0.003	0.002	−0.011
	Never										
	Seldom *	0.024	0.032	0.019	−0.020	−0.056	0.018	0.027	0.022	0.008	−0.075
**Organic**	Sometimes *	−0.013	−0.019	−0.013	0.007	0.039	−0.028	−0.053	−0.057	−0.086	0.224
	Often *	0.012	0.016	0.010	−0.009	−0.028	−0.028	−0.050	−0.051	−0.062	0.190
	Always *	−0.002	−0.004	−0.002	0.002	0.007	−0.035	−0.068	−0.076	−0.135	0.314
	Absol. not important										
**Origin**	Not important *	−0.016	−0.026	−0.018	0.007	0.053	−0.008	−0.013	−0.012	−0.009	0.042
	Indifferent *	−0.040	−0.072	−0.058	−0.015	0.185	−0.025	−0.045	−0.046	−0.060	0.175
	Important *	−0.070	−0.102	−0.070	0.028	0.215	−0.056	−0.087	−0.080	−0.070	0.293
	Very important *	−0.082	−0.111	−0.072	0.048	0.217	−0.070	−0.101	−0.088	−0.055	0.314
	TFI	0.039	0.055	0.036	−0.027	−0.103	0.023	0.035	0.031	0.017	−0.107

* The marginal probability is for a discrete change in a binary variable from 0 to 1.

**Table 7:** Regression of willingness to try food with upcycled ingredients and superior nutrition properties (TNUT) by generations.

		Generation Z (*n*= 215)		Millennials (*n*= 267)		Generation X (*n*= 264)		Baby Boomers+ (*n*= 106)	
		Coeff	*p*-Val	Coeff	*p*-Val	Coeff	*p*-Val	Coeff	*p*-Val
***Demography***			*0.015*		*0.661*		*0.651*		*0.316*
Female respondent	Genb	−0.185	0.221	0.162	0.262	0.140	0.319	−0.085	0.724
Age of respondent	Age	−2.182	0.109	0.089	0.753	−0.341	0.359	0.507	0.075
Age squared	age2	0.053	0.088	−0.001	0.732	0.004	0.362	−0.004	0.071
***Education***			*0.679*		*0.591*		*0.186*		*0.183*
Elementary school									
Middle school	ed2	−0.056	0.960	0.786	0.229			−1.507	0.033
High school	ed3	−0.171	0.383	−0.017	0.955	−0.775	0.071	−1.462	0.046
College	ed4			−0.030	0.918	−0.682	0.123	−1.496	0.034
***Working status***			*0.015*		*0.560*		*0.000*		*0.626*
Worker									
Unemployed	w2	−0.151	0.820	0.191	0.414	0.186	0.633	−0.737	0.429
Student	w3	0.571	0.351	0.318	0.253	8.978	0.000		
Homemaker	w4			−0.309	0.599	0.817	0.014	0.511	0.531
Retired	w5							−0.278	0.424
***Organic***			*0.300*		*0.148*		*0.064*		*0.493*
Never									
Seldom	bb2	−0.130	0.497	−0.370	0.024	−0.446	0.022	0.288	0.295
Sometimes	bb3	0.444	0.101	−0.147	0.676	0.127	0.639	0.111	0.881
Often	bb4	0.163	0.468	−0.128	0.539	−0.301	0.106	0.077	0.824
Always	bb5	0.209	0.437	−0.537	0.093	0.062	0.791	0.512	0.115
***Origin***			*0.874*		*0.441*		*0.000*		*0.115*
Absol. not important									
Not important	bo2	0.024	0.979	0.876	0.231	−0.601	0.207	0.129	0.932
Indifferent	bo3	−0.230	0.760	1.225	0.081	0.501	0.214	0.717	0.616
Important	bo4	−0.117	0.875	1.170	0.087	0.592	0.123	1.265	0.375
Very important	bo5	−0.288	0.698	1.110	0.109	0.870	0.027	1.327	0.337
Technophobia	TFI	−0.457	0.000	−0.525	0.000	−0.255	0.002	−0.337	0.002
									
R2		0.072		0.100		0.075		0.094	

**Table 8:** Regression of willingness to try food with upcycled ingredients and lower environmental impact (TENV) by Generations.

		Generation Z		Millennials		Generation X		Baby Boomers+	
		Coeff	*p*-Val	Coeff	*p*-Val	Coeff	*p*-Val	Coeff	*p*-Val
***Demography***			*0.852*		*0.624*		*0.570*		*0.425*
Female respondent	Genb	0.013	0.938	0.005	0.976	−0.041	0.802	−0.082	0.738
Age of respondent	Age	−0.760	0.585	−0.360	0.194	−0.162	0.694	0.032	0.912
Age squared	age2	0.019	0.564	0.005	0.199	0.002	0.657	−0.001	0.795
***Education***			*0.084*		*0.001*		*0.658*		*0.167*
Elementary school	*(omitted)*								
Middle school	ed2	−0.844	0.038	1.266	0.192			−0.277	0.598
High school	ed3	−0.403	0.068	0.988	0.001	−0.251	0.608	0.135	0.809
College	ed4			1.151	0.000	−0.369	0.461	−0.473	0.353
***Working status***			*0.007*		*0.436*		*0.000*		*0.500*
Worker									
Unemployed	w2	0.948	0.177	−0.178	0.406	0.379	0.376	−0.931	0.330
Student	w3	1.265	0.002	0.218	0.442	8.193	0.000		
Homemaker	w4			−0.529	0.239	0.827	0.012	1.177	0.297
Retired	w5							0.081	0.816
***Organic***			*0.004*		*0.013*		*0.000*		*0.023*
Never									
Seldom	bb2	−0.133	0.479	−0.320	0.082	−0.617	0.001	0.097	0.725
Sometimes	bb3	0.635	0.046	0.798	0.018	0.342	0.086	0.907	0.101
Often	bb4	0.602	0.007	0.261	0.240	0.564	0.025	1.560	0.001
Always	bb5	0.596	0.070	0.217	0.412	1.831	0.000	0.466	0.330
***Origin***			*0.057*		*0.450*		*0.000*		*0.000*
Absol. not important									
Not important	bo2	1.241	0.029	1.206	0.203	−1.585	0.004	0.706	0.486
Indifferent	bo3	0.855	0.024	1.381	0.140	−0.754	0.101	1.337	0.188
Important	bo4	1.064	0.004	1.512	0.096	−0.174	0.685	2.282	0.030
Very important	bo5	1.016	0.006	1.536	0.093	0.059	0.891	2.321	0.020
Technophobia	TFI	−0.366	0.000	−0.313	0.000	−0.274	0.001	−0.461	0.000
									
R2		0.082		0.077		0.161		0.179	

**Table 9:** Summary of the findings of the generation analysis *.

>	Full Sample		Generation Z		Millennials		Generation X		Baby Boomers+	
Var. Group	TNUT	TENV	TNUT	TENV	TNUT	TENV	TNUT	TENV	TNUT	TENV
Demography	✗	✗	✓✓	✗	✗	✗	✗	✗	✗	✗
Education	✗	✗	✗	✓	✗	✓✓	✗	✗	✗	✗
Emp. Status	✗	✗	✓✓	✓✓	✗	✗	✓✓	✓✓	✗	✗
Organic	✗	✓✓	✗	✓✓	✗	✓✓	✓	✓✓	✗	✓✓
Origin	✓✓	✓✓	✗	✓	✗	✗	✓✓	✓✓	✗	✓✓
Technophobia	✓✓	✓✓	✓✓	✓✓	✓✓	✓✓	✓✓	✓✓	✓✓	✓✓

* The null hypothesis that all coefficients in the variable group are jointly equal to zero was: (✗) not rejected, (✓) rejected at 90% confidence level, (✓✓) rejected at 95% confidence level in an χ^2^ test.

## Data Availability

The data supporting the findings of this study are available from the corresponding author (M.A.P.), upon reasonable request.
